# Circadian rhythms and cancers: the intrinsic links and therapeutic potentials

**DOI:** 10.1186/s13045-022-01238-y

**Published:** 2022-03-04

**Authors:** Li Zhou, Zhe Zhang, Edouard Nice, Canhua Huang, Wei Zhang, Yong Tang

**Affiliations:** 1 State Key Laboratory of Biotherapy and Cancer Center, West China Hospital, and West China School of Basic Sciences and Forensic Medicine, Sichuan University, and Collaborative Innovation Center for Biotherapy, Chengdu, 610041 China; 2 Department of Biochemistry and Molecular Biology, Monash University, Clayton, VIC 3800 Australia; 3 School of Basic Medical Sciences, Chengdu University of Traditional Chinese Medicine, Chengdu, 611137 China; 4 Mental Health Center and Psychiatric Laboratory, State Key Laboratory of Biotherapy, West China Hospital, Sichuan University, Chengdu, 610041 China; 5 West China Biomedical Big Data Center, West China Hospital, Sichuan University, Chengdu, 610041 China; 6 Acupuncture and Tuina School, Chengdu University of Traditional Chinese Medicine, Acupuncture and Chronobiology Laboratory of Sichuan Province, Chengdu, 610075 China

**Keywords:** Circadian rhythm, Cancer, Sleep–wake, Eating–fasting, Activity–rest, Cancer therapy

## Abstract

The circadian rhythm is an evolutionarily conserved time-keeping system that comprises a wide variety of processes including sleep–wake cycles, eating–fasting cycles, and activity–rest cycles, coordinating the behavior and physiology of all organs for whole-body homeostasis. Acute disruption of circadian rhythm may lead to transient discomfort, whereas long-term irregular circadian rhythm will result in the dysfunction of the organism, therefore increasing the risks of numerous diseases especially cancers. Indeed, both epidemiological and experimental evidence has demonstrated the intrinsic link between dysregulated circadian rhythm and cancer. Accordingly, a rapidly increasing understanding of the molecular mechanisms of circadian rhythms is opening new options for cancer therapy, possibly by modulating the circadian clock. In this review, we first describe the general regulators of circadian rhythms and their functions on cancer. In addition, we provide insights into the mechanisms underlying how several types of disruption of the circadian rhythm (including sleep–wake, eating–fasting, and activity–rest) can drive cancer progression, which may expand our understanding of cancer development from the clock perspective. Moreover, we also summarize the potential applications of modulating circadian rhythms for cancer treatment, which may provide an optional therapeutic strategy for cancer patients.

## Background

It is well known that the rotation of the Earth is a near-24-h period which endows most organisms with an internal time-keeping system to adapt to the environmental changes. This evolutionarily conserved time-keeping mechanism termed circadian rhythm allows organisms to accommodate the changing environment, such as sleep and wake in animals and opening and closing of plant flowers [1]. As early as 1729, the French astronomer de Mairan firstly reported the endogenous rhythms associated with persistent movements of plant leaves even in constant darkness. He extended his observations to patients with sleep disorders in human beings [2]. However, since these initial observations, research on circadian rhythms was minimal until the discovery of circadian genes in *Drosophila* in the 1970s which opened the door for the in-depth understanding of circadian clocks [3, 4]. In the 1990s, an explosion of data in this field contributed to the canonical action model of the circadian clock in which the Clock/Bmal1 and Per/Cry complexes play the central role [5–9]. Although the components of circadian rhythms are not conserved between species, the basic mechanisms are universal in almost all models which comprise various biological processes including anabolism and catabolism, cell division and cell cycle, immune cell functions, apoptosis and DNA damage repair [10]. Perturbation of circadian rhythms by environmental disturbances (e.g., shift work, jet lag) has been implicated in multiple pathological conditions, such as cardiovascular diseases, sleep disorders, neurodegenerative disorders, and cancers [11–13].

Several key biological processes are regulated by circadian rhythms. Thus, the disruption of the circadian clock may contribute to abnormal cell proliferation, increased gene mutation, and resistance to apoptosis, which are important hallmarks of cancer [14, 15]. Based on the findings from epidemiology and laboratory studies, abnormal circadian rhythms have been listed as a potential carcinogen by the World Health Organization (WHO), which has increased the focus on defining the underlying mechanisms of circadian disruption-induced tumorigenesis [16, 17]. Indeed, accumulating evidence has demonstrated that altered circadian rhythms are closely related to tumorigenesis in breast cancer, prostate cancer, colorectal cancer (CRC), pancreatic adenocarcinoma, liver cancer, lung cancer, and others [18–25]. In addition, therapeutic efficiency in cancer treatment is also partially dependent on the time of drug administration, which could be a new therapeutic chronotherapy strategy in cancer management [26, 27]. The intrinsic links between circadian rhythms and tumorigenesis have therefore raised interest in manipulating these rhythms to prevent malignant transformation, to develop more efficacious therapies or novel adjuvant strategies, and ultimately improve the treatment outcome of cancer patients.

In this review, we will introduce the mechanism of disruptions of circadian rhythms-derived cancer progression and discuss the potential application of pharmacological modulation of the circadian clock and/or treating cancer using the clock as new therapeutic options for improved cancer management.

## Regulation of circadian rhythms and their functions in cancer

**Fig. 1:**
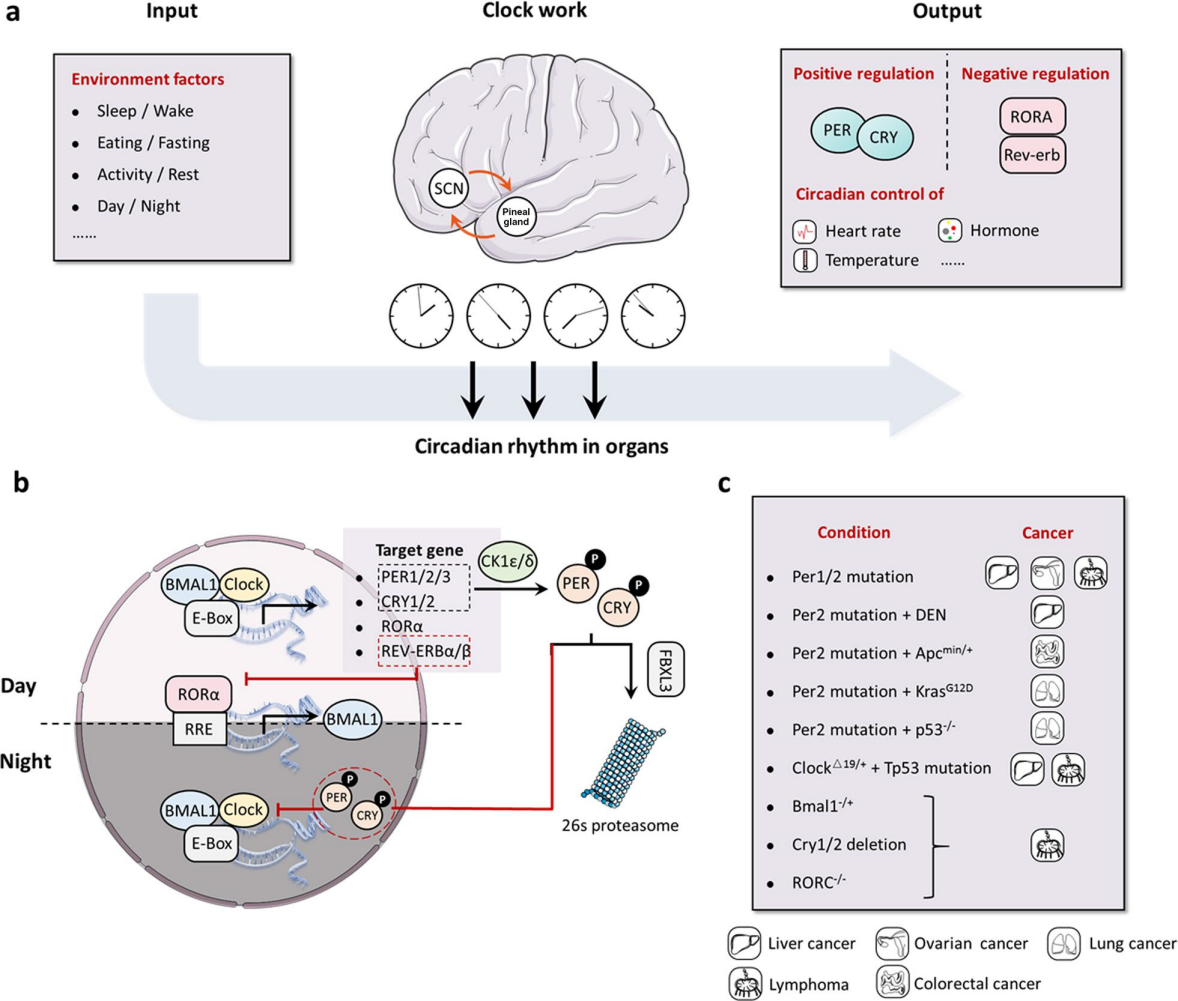
Regulation of circadian rhythms and their functions in cancer. **a** the master regulator of circadian clock is located in the suprachiasmatic nucleus (SCN) of the hypothalamus. The SCN coordinates several organ clocks in response to the environmental factors (including sleep/wake, eating/fasting, activity/rest, etc.), for controlling body homeostasis, such as heart rate, body temperature, and hormone levels. **b** at the molecular level, CLOCK and BMAL1 can bind to the CRY and PER genes through their E-boxes, thus positively regulating circadian transcription. But the mammalian CRY and PER proteins, as a heterodimer interacting with CKIε, perform a negative effect for CLOCK/BMAL1-driven transcription. In addition, the expression of circadian genes is transcriptionally modulated by the RORs and REV-ERB, resulting in the activation or repression of gene transcription for several clock genes. **c**, the mutation or deletion of core clock genes (including *Per1/2*, *Clock*, *Bmal1*, *Cry1/2* and *Rorc*) can accelerate the development of various tumors, such as liver, ovarian, lung, and colorectal cancer, and lymphoma. CLOCK: circadian locomotor output cycles kaput; BMAL1: brain and muscle aryl hydrocarbon receptor nuclear translocator 1; CRY: cryptochrome; PER: period; CKIε: casein kinase Iε

In almost all models, the signaling pathways regulating circadian rhythms are universal regardless of non-conservation of the clock components [28]. In mammals, the suprachiasmatic nucleus (SCN) of the hypothalamus holds the “master clock”, which clocks with the environmental light cycle by directly coordinating the autonomic nervous system efferent and neuroendocrine signals [29–31]. In detail, several proteins involved in the clockwork of the cell associate closely with the negative and positive transcriptional feedback loops (Fig. 1). For example, circadian locomotor output cycles kaput (CLOCK) and brain and muscle aryl hydrocarbon receptor nuclear translocator 1 (BMAL1), which contain two basic helix-loop-helix domains, can bind to the Cryptochrome (*Cry*) and Period (*Per*) genes through their E-boxes, thus positively regulating circadian transcription [32–34]. In contrast, the mammalian CRY and PER proteins, as a heterodimer interacting with casein kinase Iε (CKIε), perform a negative effect for CLOCK/BMAL1-driven transcription [35–38]. In addition, the expression of circadian genes is transcriptionally modulated by the RORs (subfamilies of nuclear hormone receptors) and REV-ERB, resulting in the activation or repression of gene transcription for several clock genes [39–42].

Growing evidence from both epidemiological research and preclinical data based on animal models supports the relationship between chronic disruption of the circadian clock and the occurrence of cancer [43–47]. Indeed, circadian disruption was listed as a probable human carcinogen in 2007 by the International Agency for Research on Cancer (IARC), part of the WHO [48].

For example, several studies have revealed that shift workers have a higher risk of developing breast and prostate cancers, as evidenced by a strong correlation between a long period (> 20 years) of shiftwork and increased cancer risk [49–52]. Furthermore, previous studies have indicated that people in modern society have undergone a sub-health lifestyle change, including excessive calories intake at midnight or continuous caloric intake over the 24 h throughout days, which mimics aspects of shiftwork and potentially promotes prostate and breast cancer risk [53, 54].

Interestingly, rest/activity rhythm was also identified as a key factor due to the significant impact on the clinical treatment of metastatic CRC patients, who have a poor quality of life with erratic periods of rest/activity [55, 56]. In line with this, the disruption of the core clock *Per2* gene further aggravated CRC cancer cell proliferation in Apc^Min/+^ genetic mice [57]. In addition, disruption of *Per2* also accelerated *Kras*^*G12D*^ and *Kras*^*G12D*^/*p53*^−/−^ mutation-mediated lung cancer progression [23].

Additionally, mice with the core clock *Per2* genes disrupted were more tumor-prone following treatment with the carcinogenic agent diethylnitrosamine (DEN) [58, 59]. Moreover, irradiation stimuli promoted the development and progression of liver cancer and lymphoma in *Per1* and *Per2* mutant mice and *Bmal1*^*−/*+^ mice [60]. Mice with deleted *Cry1* and *Cry2* or *Rorc* showed similar effects in lymphoma [60, 61]. There has also been a report on a link between *Clock* mutation *Clock*^*Δ19/*+^ and *Tp53* mutation-induced liver cancer and lymphoma [62].

However, some studies demonstrated that clock gene mutations (including *Clock*, *Per1*^*−/−*^ or *Per2*^*−/−*^, *Cry1*^*−/−*^ and *Cry2*^−/−^) potentially led to an adverse influence on cancer progression [63, 64]. In a RAS mutation-triggered cutaneous squamous tumor model, *Bmal1* deletion protects from tumorigenesis [65]. In addition, previous studies have revealed CLOCK and BMAL1 as key regulators for acute myeloid leukemia (AML) by contributing to proliferation and stemness [66, 67]. Moreover, deletions of *Cry1* and *Cry2* diminished tumor development in *p53* null mice possibly due to triple mutant-induced genotoxic stress [68, 69]. Taken together, the dual function of clock genes, either as oncogenes or as tumor suppressors, may be attributed to tissue-specific mechanisms and implies the existence of intricate clock-dependent networks, which are involved in the maintenance of homeostasis during different tumor stages.

In summary, these rhythms mainly emerge from the interplay between circadian clocks and sleep–wake cycles, eating–fasting cycles, and activity–rest cycles. These circadian rhythms are able to modulate several key aspects of cellular and organ functions with profound implications in cancer management. In the following sections, we will discuss the interplay between these cycles with cancer and describe the links involved.

## The interplay between sleep–wake cycles and cancer

**Fig. 2:**
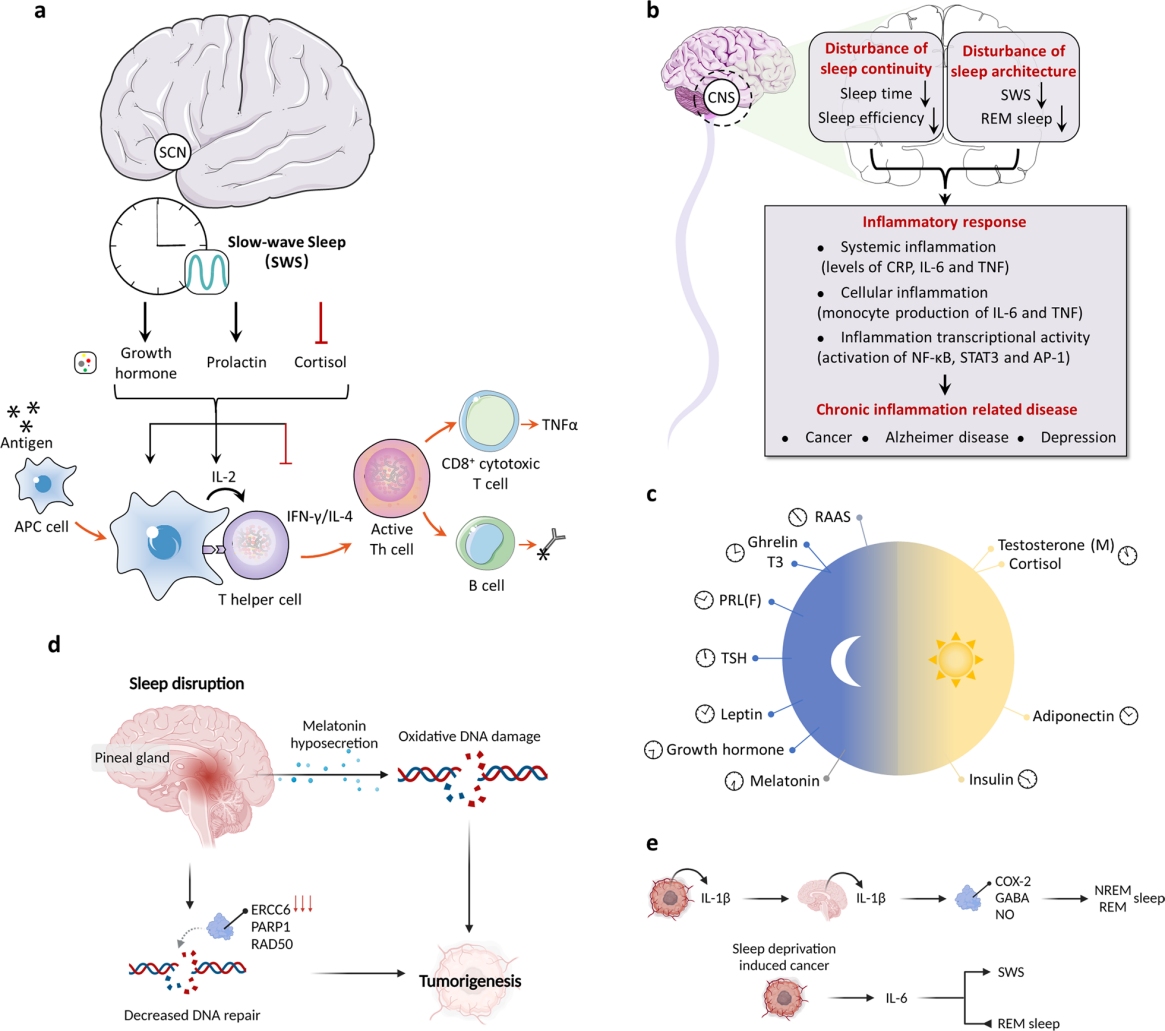
The interplay between sleep–wake cycles and cancer. **a** sleep–wake cycles regulate the immune system. During normal sleep–wake cycles, SWS sustains the function of immune system by maintaining the balance of T-helper1 (Th1) and T-helper2 (Th2) cell-derived cytokines (Th1 cytokines: IL-2, IFNγ, and IL-12; Th2 cytokines: IL-4 and IL-10), which benefit the antigen presenting process. **b** sleep–wake cycles regulate the inflammatory response. Disturbance of sleep continuity (sleep time and efficiency) and architecture (SWS and REM sleep) may lead to body inflammatory response, including abnormal systemic inflammation, cellular inflammation and inflammation transcriptional activity, which are associated with development of chronic inflammation related disease, such as cancers. **c** Sleep–wake cycles and endocrine. Endocrine factors, including growth hormone, prolactin, thyroid hormone, cortisol, gonadal steroids, insulin, and so on, have been identified to be secreted during certain time periods. Disruption of sleep–wake cycles may break these balances and influence their secretions. **d** Sleep–wake cycles regulate DNA damage and repair. On the one hand, sleep disruption can reduce the levels of melatonin, an important antioxidant, which may lead to increased oxidative DNA damage. On the other hand, sleep deprivation downregulates the expression of several genes involved in DNA repair, such as ERCC6, PARP1, and RAD50, which may ultimately promote tumorigenesis. **e**, Feedback from cancer to sleep–wake cycles. IL-1β in the brain can regulate REM and NREM sleep by modulating various molecules and neurotransmitters, including COX-2, GABA, and nitric oxide (NO), while IL-6 enhances SWS and decreases REM sleep. SWS: slow-wave sleep; REM: rapid eyes movement; TSH: thyroid-stimulating hormone; PRL: prolactin; RAAS: renin–angiotensin–aldosterone systems; COX-2: cyclooxygenase-2.

Disruption of sleep–wake circadian rhythm has a major impact on the entire neuroendocrine-immune system, which modulates immune defense and energy metabolism, thus contributing to normal physiological activity by coordinating the biological response to everyday stresses, as well as cognitive and physical performance (Fig. 2).

### Sleep–wake cycles regulate the immune system

Rhythmical biological regulation of the immune system is mediated by intricate crosstalk between the nervous and endocrine systems [70–72]. Dysregulation of sleep–wake cycles, like prolonged sleep deprivation, disrupts the immune system, resulting in increased rates of viral infection and weak antigen processing and antibody formation [70, 73, 74].

During normal sleep–wake cycles, the immune system displays a biphasic shift that is linked with maintaining the balance of T-helper1 (Th1) and T-helper2 (Th2) cell-derived cytokines (Th1 cytokines: IL-2, IFNγ, and IL-12; Th2 cytokines: IL-4 and IL-10) [75]. Mechanically, Th1 activity in the initial hours of nighttime sleep is enhanced and accompanied by moderate promotion of interferon (IFN)-γ/IL-4-producing Th cells, while Th2 activity dominates in the late hours of sleep or before wake up [76]. Once this pattern is broken, cytokines are excessively produced and directly induce immune disturbances, which subsequently lead to chronic inflammation and tissue damage. In sleep-deprived individuals, including stressed persons, insomniacs, and the elderly, a switch toward Th2 activity has been reported [76]. Sleep deprivation-induced production of growth hormone and prolactin turning cytokine balance into Type 1 dominance, as well as cortisol and norepinephrine leading to Type 2 dominance, are all involved in immune response alteration [70, 76, 77].

In addition, decreased cellular immunity is also attributed to the elevation of sympathetic tone induced by sleep deprivation. During sleep, the number of monocytes producing IL-12 or IL-10 is normally increased or decreased, respectively, thus resulting in a circadian rhythm. However, when chronic wakefulness occurs, rhythmic temporal variations of IL-12 and IL-10 produced from monocytes are lost, causing dysregulation of the immune system [77]. Furthermore, Irwin and colleagues revealed that the lytic activity of natural killer (NK) cell was diminished by an average of 28% in people with sleep deprivation, i.e., wakefulness between 03:00 to 07:00 h, compared with blood sampling between 07:00 and 09:00 h from healthy men [78]. They then demonstrated that partial sleep deprivation potentially attenuates the natural immune response, evidenced by decreased numbers and weak lytic activity of NK cells, lymphokine-activated killer (LAK), and its precursors [79]. Notably, a marked decrease of CD8^+^ T cells (cytotoxic and memory), which are derived from tumor necrosis factor (TNFα) stimuli, is the most pronounced effect resulting from sleep deprivation, solidly implying an impaired immune response if exposed to carcinogenic risk [76]. These findings suggest that even modest disruption of sleep–wake cycles resulting from working the night shift can significantly induce the dysregulation of natural immune responses, T cell-cytokine production, etc. These dysregulations need more than a single day of sleep to recover to baseline and potentially lead to an increased risk of cancer [80–82].

### Sleep–wake cycles regulate inflammatory responses

Inflammatory responses are abnormally activated following dysregulation of the immune system caused by disruption of sleep–wake cycles, which is characterized by the activation of soluble intercellular adhesions molecule (sICAM)-modulated NF-κB inflammatory signaling and the subsequent induction of inflammatory markers TNF-γ, IL-6, and C-reactive protein (CRP) [83, 84]. In addition, sleep deprivation alters IL-6-mediated trans-signaling by changing the levels of soluble IL-6 (sIL-6) receptor in various types of brain cells and neighboring organs [85]. An analysis using a DNA microarray indicated that sleep-deprivation-induced upregulation of inflammatory genes in humans was a common response and might be not rapidly resolved [83]. Sleep restriction-triggered upregulation of proinflammatory cytokines (IL-6, IL-1β, IL-17, and CRP) were maintained at high expression levels even through two nights of recovery sleep [86]. In line with this, increased levels of CRP were also found in shift workers, indicating a progression of an inflammatory state and an increased risk of cancer [87].

Rigorous studies profiling inflammatory markers in the human sleep–wake cycle are limited to a few methodological studies that have looked at profiles of systemic and cellular inflammatory markers obtained repeatedly over the course of a regular sleep–wake cycle, compared with continuous wakefulness over 24 h [88]. These studies have shown a close association between the levels of several markers and sleep, the circadian oscillator, or both [70]. For example, IL-6 was reported to have a circadian profile whose levels peaked at 19:00 h and 05:00 h [89]. Furthermore, upregulation of IL-6 levels and production of TNF was attributed to nocturnal sleep [70]. A previous study indicated that experimental sleep deprivation during the nocturnal period resulted in an undersecretion of IL-6 and an attenuated secretion of TNF from Toll-like receptor 4 (TLR4)-induced monocytes [90]. Notably, sleep deprivation changed the nighttime pattern of IL-6 secretion into daytime, therefore resulting in an IL-6 oversecretion during the day [89]. Similarly, by investigating people with sleep disturbances, TLR4-mediated IL-6 production from monocytes was also found to be decreased at night and increased in the day [91]. Nevertheless, IL-6 levels were partially mediated by the circadian oscillator as indicated by the observation of transient (less than 1 h) peaks during nocturnal wakefulness [85].

Different stages of sleep, including slow-wave sleep (SWS) and rapid eyes movement (REM) sleep, differentially induced nocturnal changes of inflammatory cytokine activity. However, the levels of the inflammatory cytokines during the detailed period were not clear. The levels of inflammatory cytokines were reported to peak early in SWS, but higher levels of IL-6 were found during REM sleep compared with being awake [92]. Consistent with increased IL-6 during REM sleep, IL-6 and sIL-6R were upregulated later during the night, and the morning levels of TLR4-stimulated IL-6 production from monocytes were found to correlate with the amount of REM sleep [85, 91, 92].

In conclusion, sleep disruption may lead to abnormal inflammatory responses as evidenced by the upregulation of proinflammatory cytokines (especially IL-6 and CRP), which are potential risk factors for several cancers, including ovarian, brain, breast and colorectal cancers.

### Sleep–wake cycles and endocrine factors

Endocrine factors, especially those governed by the hypothalamic-pituitary axis, have been identified to be regulated by circadian rhythms, therefore fluctuating over the day. These include growth hormone (GH), prolactin (PRL), thyroid hormone, cortisol, and gonadal steroids [93–95]. Insulin and adipokines, a form of nutrient-sensitive hormones, are also mediated by circadian rhythms (light/dark and feeding/fasting cycles) giving rise to varying circulating levels, partially controlled by time-of-day-dependent patterns [93].

Growth hormone and prolactin have been reported to change their secretory pattern due to exposure to acute sleep deprivation [96]. This treatment also mitigated nighttime peaks of growth hormone and prolactin, leading to dysregulation of the immune system [70]. Furthermore, a previous study demonstrated that the 24-h mean and amplitude of several endocrine factors modulated by the circadian clock, including thyroid-stimulating hormone, prolactin, and leptin, were suppressed by semi-chronic sleep deprivation with sleep debt during 6 days [93]. Sleep debt also influenced the circadian pattern of plasma cortisol, resulting in higher plasma levels in the afternoon and early evening [93].

Notably, hyperphagia and weight gain are closely associated with sleep deprivation. Mechanically, sleep deprivation triggers downregulation of the plasma concentration of the appetite-restraining adipokine leptin and upregulation of the plasma concentration of the appetite-stimulating peptide ghrelin, in which adipokine leptin and peptide ghrelin are produced in adipose tissue and the stomach, respectively [97]. Accordingly, this process leads to greater hunger and appetite, subsequently resulting in triglyceride metabolism and disturbed lipid levels [98]. Inhibition of appetite is required for the secretion and blood levels of leptin, which are positively regulated by daily sleep length and display significant reduction with sleep deprivation [97, 99]. Indeed, overweight or obesity risk in shift workers is found to have a potential relationship with sleep deprivation-induced changes of the hormonal milieu. Numerous studies have comprehensively revealed that weight gain or various levels of obesity, defined by increased body mass index and/or elevated waist-hip ratio, was potentially mediated by prolonged shift work-induced circadian disruption [100, 101]. In addition, marked weight gain was also confirmed, even 4–5 years after shift work exposure in prospective cohort studies [102]. However, weight modulation in shift work is a cumulative effect because it is also regulated by various factors, such as age, lifestyle as well as “night eating syndrome” [103]. Nevertheless, either overweight or obesity facilitated increased secretion of adipokines and release of macrophages from adipose tissue, thus triggering chronic inflammation and subsequent oxidative stress and DNA damage [104]. These events likely contribute to breast cancer promotion and poor prognosis at least in a subpopulation, for example, postmenopausal female shift workers [105].

### Sleep–wake cycles regulate DNA damage and repair

Stress-induced DNA damage has long been recognized as an important risk factor for the development of several chronic diseases, including cancer [106–108]. Sleep disruption in shift workers may lead to increased oxidative DNA damage due to decreased secretion of melatonin (an important cellular antioxidant) caused by sleep disruption [109–112]. For example, urine samples from 217 dayshift workers and 223 nightshift workers were collected for analysis of 8-OH-dG (a marker of oxidative DNA damage) and aMT6s (a marker of circulating melatonin) levels by using high-performance liquid chromatography with electrochemical detection [113]. The results found that disruption of melatonin in nightshift workers is associated with impaired DNA repair machinery and increased cellular oxidative DNA damage, as evidenced by the positive correlation between aMT6s and urinary 8-OH-dG levels [113]. Another study also demonstrated that night work is associated with reduced DNA repair, and that this effect was probably due to melatonin suppression caused by night work, which raises the potential of melatonin supplementation for preventing DNA damage-induced chronic diseases [114]. Moreover, an observational study on 49 healthy doctors has found that nightshift doctors with sleep deprivation showed lower expression of DNA repair genes and increased DNA breaks compared with the dayshift colleagues, which was linked to the development of chronic disease [115]. In a recent controlled laboratory study, transcriptomic analysis of genes and associated pathways in circulating leukocytes obtained from night shift workers and healthy adults demonstrated a significantly impaired DNA repair pathway, which increased DNA damage and cancer risk in shift workers [116]. Taken together, these findings indicate that chronic disruption of sleep–wake cycles may cause increased DNA damage and decreased DNA repair, serving as potential risk factors for tumorigenesis [117–119].

### Feedbacks from cancer to sleep–wake cycles

Various cytokines, such as TNF-α, TGF-β, IL-10, and especially IL-1β and IL-6, are well studied and have been shown to be involved in the complex crosstalk between cancer initiation/progression and sleep–wake cycles [120, 121].

Notably, IL-1β can not only act as a pleiotropic cytokine to promote cancer progression in the tumor microenvironment but can also gain access to the brain through passive diffusion, subsequently interacting with IL-1R1 expressed on endothelial cells at the blood–brain barrier or vagal afferents [122–124]. Accordingly, IL-1β in the brain has been identified as the key mediator to affect rhythmical behavior. A previous study reported that both duration and delta power (~ 0.5–4 Hz oscillations) of NREM (non-rapid eye movement) sleep were markedly enhanced by injecting central or systemic IL-1β, which might play a role in hypnosis [125]. In contrast, spontaneous NREM sleep could be inhibited by the administration of IL-1β receptor antagonists or IL-1β neutralizing antibodies [126, 127]. For REM sleep, IL-1β regulates it in a time and dose-dependent manner, shown by high levels of IL-1β inhibiting REM sleep, while low levels of IL-1β have no effect [121]. Moreover, IL-1β can influence sleep by modulating various molecules and neurotransmitters, including cyclooxygenase-2, NF-κB, GABA, nitric oxide (NO), prostaglandins, and adenosine [128]. For instance, inhibition of NO synthesis by an inhibitor, L-NAME, could decrease NO production and IL-1β-induced NREM sleep [129]. Notably, sleep deprivation could in turn stimulate the expression of IL-1β in the brain [130]. Overall, these studies suggest that IL-1β is under circadian and homeostatic control and has effects on multiple sleep nuclei.

IL-6 has been suggested to follow diurnal rhythms, but its detailed role is not yet totally clear [121, 131]. Like IL-1β, IL-6 during wakefulness is normally at low levels and peaks during sleep [89]. Sleep deprivation elevates plasma levels of circulating IL-6 [92]. A study in humans revealed that IL-6 enhanced SWS and decreased REM sleep following subcutaneous injection [132]. Paradoxically, human recombinant IL-6 injected intracerebroventricularly into rabbits seemed to be not somnogenic but pyrogenic [133]. However, recombinant rat IL-6 reduced NREM sleep after temporarily enhancing NREM sleep in rat models [134]. In fact, the relationship between IL-6 and sleep–wake cycles is intricate. For example, sleep enhanced the levels of sIL-6R to over 70% of the wake levels at the termination of sleep, and simultaneously induced IL-6 trans-signaling regardless of classical/membrane-bound IL-6 signaling [85]. This process further supports the role of sleep in immune defense [135, 136].

In summary, the above findings suggest that the disruption of sleep–wake cycles may serve as a potential risk factor for tumors mainly through modulating the neuroendocrine-immune system, the inflammatory responses, and the DNA damage repair systems. Moreover, increased levels of inflammatory cytokines secreted from tumors or tumor-associated leukocytes could in turn control the homeostasis of several sleep process, implying the complex crosstalk between cancers and sleep–wake cycles. Therefore, maintaining regular sleep pattern is highly encouraged in daily life for prevention of not only cancer but also other disorders.

## The interplay between eating–fasting cycles and cancer

**Fig. 3:**
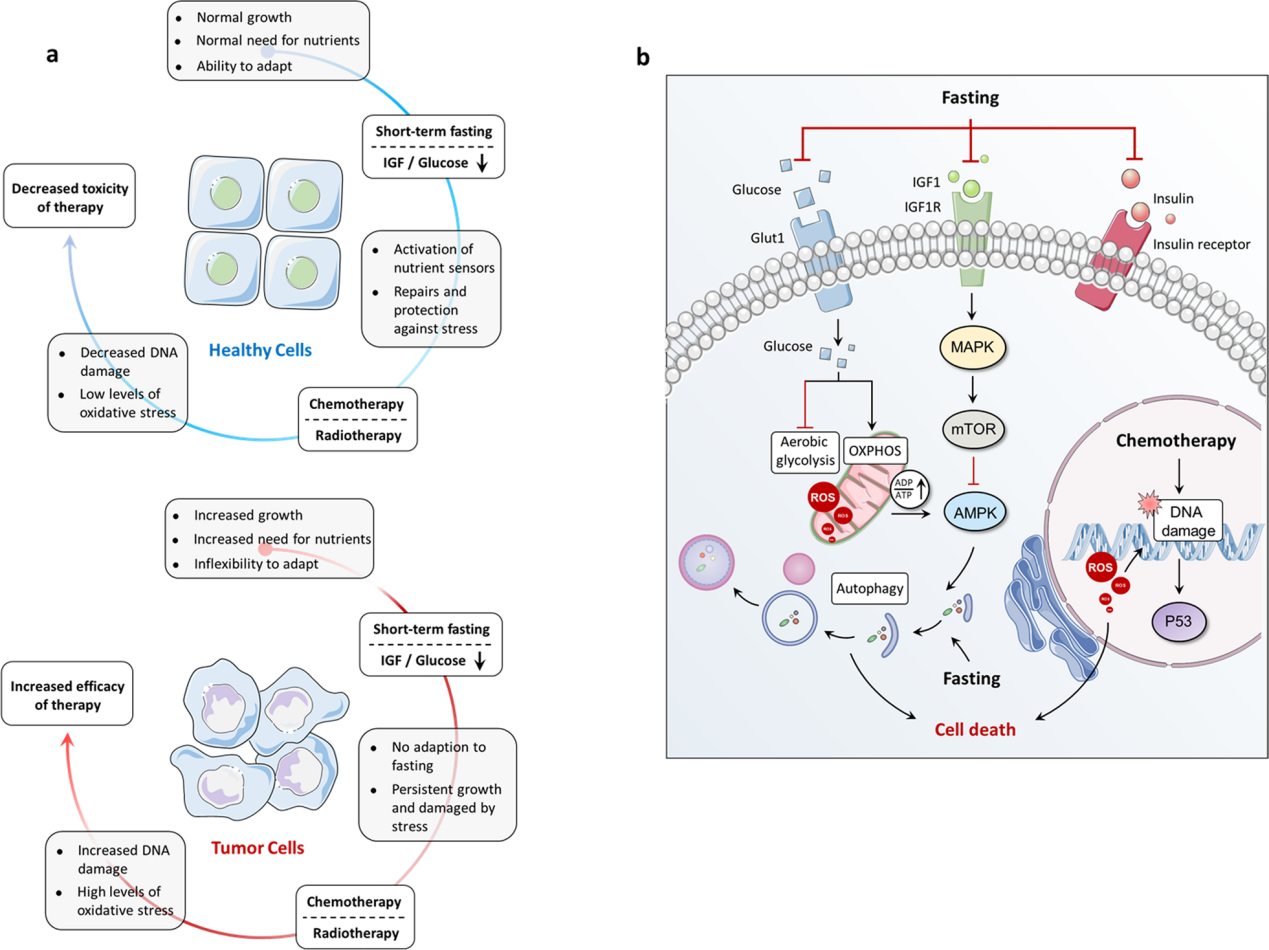
The interplay between eating–fasting cycles and cancer. **a** eating–fasting cycles regulate differential stress resistance and sensitization of cancer cells. Fasting triggers metabolic reprogramming of adipose tissue and muscle, which on the one hand sensitizes cancer cells to chemotherapy and other cancer therapies while on the other protects healthy cells from side effects from tumor therapy. **b** fasting or FMDs is able to decrease the levels of growth-promoting nutrients and factors, including glucose, IGF1 and insulin. Decreased glucose levels coupled with reduced glucose uptake via inhibition of GLUTs result in downregulated aerobic glycolysis and increased OXPHOS. This metabolic switch increases the accumulation of cellular ROS levels in cancer cells in response to chemotherapy, resultantly causing oxidative DNA damage and cell death. In addition, fasting or FMDs can also regulate IGF1R-mTOR-AMPK signaling to activate anticancer immunity by inducing autophagy. FMD: fasting-mimicking diets; GLUT: glucose transporter; OXPHOS: oxidative phosphorylation; ROS: reactive oxygen species

Dietary-related risk factors have been identified as one of the key determinants for cancer development [137–139]. In line with this notion, 14–20% of all cancer-related mortality in the USA is associated with obesity induced by dysregulation of eating–fasting cycles, and guidelines for nutrition administration that advocate maintaining eating–fasting cycles are thus suggested for reducing cancer incidence [138, 139]. In addition, preclinical data have revealed that cancer cells with hyperproliferation usually fail to adapt to fasting conditions, implying the possibility that calorie-limited diets based on eating–fasting cycles could become a potential strategy for the prevention and treatment of cancer [140, 141]. Notably, balancing eating–fasting cycles seems to improve acute and/or chronic side effects from treatments in cancer patients. Herein, we summarize the detailed mechanisms behind eating–fasting cycles-mediated signaling pathways for cancer initiation and development, and emphasis the important role of ordered eating–fasting cycles in cancer prevention or therapy (Fig. 3).

### Effects of eating–fasting cycles on hormones and metabolites

The levels of metabolites and circulating hormones are changed in response to eating–fasting. This is characterized by downregulation of glucose, IGF1, leptin, and insulin, and upregulation of adiponectin, which contribute to either antitumor effects and/or protection from side effects [142, 143]. Increasingly strong evidence is demonstrating that fasting or fasting-mimicking diets (FMDs) inhibits the effect of IGF1 and glucose against cancer [144]. Mechanistically, fasting or an FMD compromises various onco-pathways, such as cAMP-PKA and IGF1R-AKT-mTOR-S6K signaling, activates anticancer immunity, and enhances the tolerance of normal cells in stress conditions by inducing autophagy [145–147]. In addition, fasting has been revealed to induce ketone body accumulation, leading to mitigation of tumor proliferation and promotion of differentiation by inhibiting histone deacetylases (HDACs) [148].

### Eating–fasting cycles regulate differential stress resistance and sensitization of cancer cells

Fasting triggers metabolic reprogramming of adipose tissue and muscle, where carbon-related metabolic pathways are modulated to change the levels of certain metabolites and circulating hormones. On the one hand these processes sensitize cancer cells to chemotherapy and other cancer therapies, while on the other they protect healthy cells from side effects from tumor therapy [140, 141].

Several studies using animal models have demonstrated that short-term fasting (STF) promoted the anticancer effects of chemotherapeutic agents against numerous cancers, including pancreatic cancer, breast cancer, colorectal cancer, melanoma, and neuroblastoma, while simultaneously diminishing toxic effects of the treatments [149–151]. Furthermore, a 24–60-h fasting regime reinforced chemotoxicity tolerance of healthy cells, and further enhanced anticancer effects of chemotherapy compared to chemotherapy alone in different strains of mice with tumor xenografts [145, 152–154]. The distinct outcomes derived from STF in healthy or tumor cells are termed differential stress resistance (DSR) [155]. Similarly, healthy cells faced with nutrient deprivation utilize energy for tissue repair that facilitates resistance to chemotherapy, while tumor cells still maintain hyperproliferation thus resulting in increased DNA damage and apoptosis during chemotherapy [141, 152, 156]. Thus, differential stress sensitization (DSS) is defined as a phenomenon whereby STF protects healthy cells from chemotherapy-induced toxicity and enhances the antitumor effect of chemotherapy.

#### Differential stress resistance (DSR)

The hypothesis that starvation plays an adverse effect in cancer compared to normal cells is reasonable, especially when the organism simultaneously faces cell stressors such as chemotherapy. In detail, healthy cells during starvation reduce global ribosome biogenesis and growth-related gene transcription which facilitates entry into the self-maintenance mode to resist damage caused by various cancer treatments. By contrast, the self-maintenance mode is terminated due to onco-pathways in cancer cells, thus leading to the reduction of stress response signaling [141]. This study also demonstrated that low-glucose triggered further cell death of mouse or human glioma and neuroblastoma cancer in hydrogen peroxide or cyclophosphamide-treated models, while protecting healthy glia cells from treatment-induced toxicity [141]. In line with these results, etoposide treatment markedly promoted the survival rate of neuroblastoma allograft-bearing mice following 2-day fasting, while resulting in only moderate effects in non-fasted mice [141].

A subsequent study indicated that fasting-triggered inactivation of IGF1 signaling protected glia and neurons from the oxidative stress agent cyclophosphamide and reduced doxorubicin-induced mouse embryonic fibroblasts, but not glioma and neuroblastoma cells [144]. Furthermore, mice with a conditional liver Igf1 gene deletion, which mimics fasting characterized by decreased levels of circulating IGF1, showed a toxicity-resistant ability against chemotherapeutic drugs, such as doxorubicin [157]. Histopathology studies suggested that compromised cardiac myopathy induced by doxorubicin was present in control mice but not in limited ingredient diet (LID)-treated mice [144]. Taken together, these data demonstrate that fasting-induced IGF1 downregulation is involved in increased tolerability to chemotherapy.

Fasting also contributes to overcoming the bottleneck for clinical application of both mTOR inhibitors and dexamethasone, which are used as anti-emetics and anti-allergics or as anticancer agents in cancer therapy, but have non-negligible side effects, such as hyperglycemia [149]. Indeed, the high glucose-induced cAMP-PKA pathway, which was attributed to both dexamethasone and rapamycin treatment, was revealed to enhance the toxicity of mouse cardiomyocytes from doxorubicin [149]. These results could possibly be reversed by decreasing circulating glucose concentration via fasting or insulin injections. In addition, fasting and/or FMD-triggered autophagy is also involved in the reduction of doxorubicin-induced cardiomyopathy by removing damaged mitochondria and toxic aggregates, therefore resulting in the reduction of reactive oxygen species (ROS) with protective effects on cardiomyocytes [158].

#### Differential stress sensitization (DSS)

Dietary treatment alone (like fasting and FMDs) shows limited effects on cancer. However, based on the DSS-derived mechanism, dietary interventions combined with canonical cancer therapy potentially display promising anticancer effects [159–161]. Previous observations on glioma, melanoma, and breast cancer indicated that fasting induced unexpected upregulation of ribosome biogenesis and expression of assembly genes or proliferation-related genes, which contribute to the activation of AKT and S6K and subsequent ROS accumulation and DNA damage, further increasing the effect of DNA-damaging drugs [140]. In fact, periodic cycles of fasting or FMDs in mice were reported to inhibit cell growth in several solid tumors and lymphoid leukemia [147, 159]. Notably, periodic fasting or FMDs were also revealed to enhance the anticancer effects of chemo- or radiotherapy and tyrosine kinase inhibitors (TKIs) [162–164].

In addition to regulating glucose utilization and fatty acid β-oxidation in cancer, fasting or FMDs can also accelerate the conversion of energy metabolism mechanisms in cancer cells from aerobic glycolysis to mitochondrial OXPHOS for maintaining the growth of cancer cells in the nutrition-deprivation environment [152]. Subsequently, mitochondrial OXPHOS promotes ROS accumulation and decreases glutathione synthesis primarily derived by glycolysis and the pentose phosphate pathway, which leads to reduced ROS-mediated DNA damage. Strategies inhibiting glycolysis and glutaminolysis and promoting OXPHOS in order to delay tumor growth and overcome drug resistance are under investigation [152]. Of note, fasting or FMDs seem to promote metastasis, since several types of aggressive and metastatic cancer cells tend to be dependent on high-lactate production from high glycolytic activity [165].

Fasting or FMDs can trigger other alterations besides the metabolism changes that induce DSS in cancer cells. For example, fasting upregulated equilibrative nucleoside transporter 1 (ENT1) expression (gemcitabine transporter) to improve the anticancer activity of gemcitabine against pancreatic cancer [166]. In breast cancer and melanoma cells, fasting could induce SUMO modification of REV1 (a p53-binding protein and DNA polymerase) by the SUMO2 and/or SUMO3-dependent ways, consequently relieving the suppressive effect of REV1 on p53, which inhibited cancer cells by transcriptionally promoting expression of the pro-apoptotic genes [151]. In addition, a fasting-mimicking diet enhances the effect of the anti-estrogens tamoxifen and fulvestrant by reducing circulating IGF1 through EGR1 and PTEN-mediated inhibition of AKT/mTOR signaling [159]. In leukemia, a previous study reported that fasting could slow the progression of acute lymphoblastic leukemia (ALL) but not acute myeloid leukemia (AML) by activating the protein PR/SET domain 1 (PRDM1)-mediated leptin receptor and downstream signaling [167]. Furthermore, transcription factors PAX5 and IKZF1, commonly identified with more than 80% mutations in pre-B cell ALL, were indicated as tumor suppressors by exhibiting a persistent restriction on glucose uptake and energy supply [168]. Accordingly, energy crisis and cell death were observed in *PAX5* and *IKZF1* reconstituted pre-B-ALL cells, indicating a potential for developing the clinical application of fasting or FMDs in ALL [168].

Taken together, these observations demonstrate that although dysregulation of eating–fasting cycles have been identified as a key determinant for cancer development, fasting or FMDs can not only increase the sensitivity of cancer therapies but also reduce the side effects from chemotherapy, indicating a promising strategy for the prevention and treatment of cancer.

## The interplay between activity–rest cycles and cancer

**Fig. 4:**
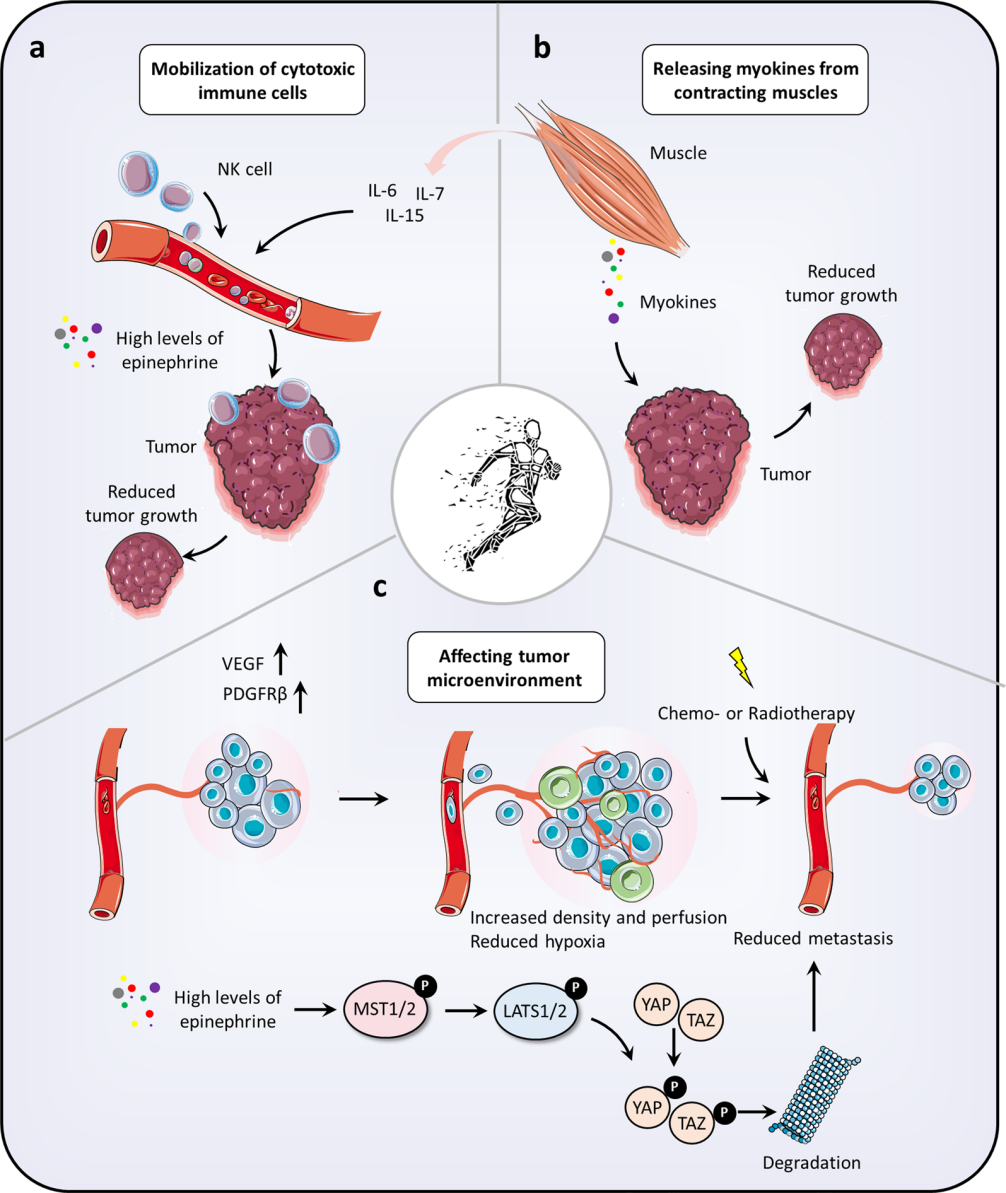
The interplay between activity–rest cycles and cancer. **a** activity–rest cycles regulate immune function for tumor suppression. Exercise has been found to promote immune cell infiltration by epinephrine-driven NK cells circulation, resulting in a marked inhibition of cancer. Moreover, exercise also enhances naïve T cell populations and mitigates detrimental effects on T cells, leading to more efficient generation of immunological memory. **b** activity–rest cycles regulate the crosstalk between muscle and tumor. The crosstalk between muscle and tumor depends on skeletal muscle-secreted myokines. Exercise-induced myokines, including OSM, irisin, and SPARC, may play a role in cancer prevention and therapy. In addition, exercise-induced myokines can also induce the secretion of immune regulatory cytokines, including IL-6, IL-7, and IL-15, which indirectly regulate immune cell function. **c**, activity–rest cycles regulate tumor microenvironment. Exercise-mediated promotion of pro-angiogenic cytokines (such as VEGF) induces vascular remodeling to increase density and perfusion and reduce hypoxia in the tumor microenvironment. Furthermore, exercise was involved in the regulation of the Hippo signaling pathway, in which exercise-driven epinephrine markedly inhibited tumor formation by inducing Yap and Taz phosphorylation and subsequent degradation. NK cells: natural killer cells; OSM: oncostatin M; SPARC: secreted protein acidic and rich in cysteine; VEGF: vascular endothelial growth factor

Based on epidemiological analyses, physical activity is associated with decreased risk of multiple cancers, including colorectal, breast, and prostate cancer [169–173]. Preclinical studies have also indicated that exercise has potential for cancer prevention (Fig. 4). Exercise training, such as treadmill running, voluntary wheel running, and swimming has been found to inhibit tumor initiation and progression in chemical, genetic or transplantable-triggered tumor mouse models [174–177]. However, exercise training has been shown to display different effects against a variety of cancers with different genetic backgrounds. For example, in the p53-deficient MMTV-*Wnt* mouse model bearing breast cancer, exercise training seemed to be ineffective against this cancer [178]. Nevertheless, strengthening physical exercise is still suggested for combating cancer regardless of cancer diagnosis. Notably, physical activity-driven antitumor effects can potentially be attributed to several mechanisms, which have different effects on different kinds of cancers.

### Activity–rest cycles modulate immune functions

Exercise can modulate the circulation of immune cells by a mechanism mediated by adrenergic signaling and blood flow-induced shear stress, facilitating immune surveillance by eradicating identified malignant cells [179]. Indeed, an increasing number of studies have found exercise-induced immune cell mobilization as a common phenomenon independent of age, gender, or tumor type [180, 181]. A study focusing on patients with breast cancer convincingly demonstrated that NK cell mobilization in breast cancer survivors during exercise was comparable to that in age-matched healthy people [182]. However, a recent randomized controlled trial suggested that exercise displayed no significant impact on resting NK cell function and circulating myokines in women with high-risk breast cancer [183], indicating a potentially ambiguous role of exercise in regulating NK cell function for cancer immunotherapy.

In terms of preclinical studies, transplantable or genetic animal cancer models are usually used to investigate the association between exercise and cancer [184, 185]. For example, exercise was found to activate the immune system to suppress tumor progression in a genetic mouse model of mammary cancer following exercise training [186]. In addition, exercise-mediated immune system activation, such as NK cells and cytotoxic T cells infiltration, was also revealed in genetic tumor models, suggesting that additional immunological response was induced in the exercise-induced effect besides the initial immune response to the inoculated tumor cells [180, 187]. In a wheel-running mouse model, exercise was revealed to promote immune cell infiltration by epinephrine-driven NK cells circulation, resulting in a marked inhibition of cancer growth [185]. Exercise also enhances naïve T cell populations and mitigates detrimental effects on T cells, leading to more efficient generation of immunological memory [188, 189].

Increased body temperature is a major outcome of exercise, promoting the function and stimulus of immune cells [190]. A well-known observation is that hyperthermia can expand the diameter of intratumor blood vessels, therefore enhancing NK cells infiltration into the tumor and inhibiting cancer cell growth [191]. Furthermore, hyperthermia accelerates cytotoxic T cell recruitment into the tumor microenvironment by IL-6 trans-signaling-mediated tumor vasculature remodeling [184]. In fact, hyperthermia has been applied to clinical treatment for certain cancers, although the therapy temperature is generally higher than if exercise training-induced [192–194].

In summary, exercise could therefore be considered for cancer prevention or therapy due to its potential to promote an anticancer immunological response. However, exercise-mediated modulation of the immune system is complex, and the detailed mechanisms need to be further investigated [195–197].

### Activity–rest cycles regulate the crosstalk between muscle and tumor

The crosstalk between muscle and tumor depends on skeletal muscle-secreted myokines produced during muscle contractions [198–201]. Recently, with the emergence of omics-based strategies, new exercise-induced myokines are gradually being identified in the muscle secretome [202–206]. Although the evidence, that exercise-driven myokines may play a role in cancer prevention or therapy, is still limited, several preclinical studies have indicated muscle-derived Oncostatin M (OSM) and Irisin may be effective against prostate and breast cancer [183, 207–210]. In addition, the myokine secreted protein acidic and rich in cysteine (SPARC) produced by exercise training has been identified as an anticancer factor, as evidenced by decreased tumorigenesis in trained mice bearing colon cancer [211–213]. Notably, myokines are divided into distinct classes, with the potential to modulate the proliferation and differentiation of cancer cells by either directly inducing cell growth or antagonizing certain ligands [208, 214–216]. Some myokines (like irisin) have been demonstrated to be modulators involved in the common cancer-related pathways, such as TGF-β or Wnt signaling [217–219].

Exercise-induced myokines can also induce the secretion of immune regulatory cytokines, including IL-6, IL-7, and IL-15, which indirectly regulate immune cell function [220–223]. For humans, IL-6 levels are controlled by exercise in a time and intensity-dependent way and are also related to the amount of muscle mass engaged in the exercise [224–226]. However, the importance of IL-6 produced during exercise in immune responses remains less well understood. A recent study indicated that exercise-mediated IL-6 seemed to improve immune cell infiltration. Anti-IL-6 antibody-mediated blockage of IL-6 signaling partially reversed the efficacy of exercise on inhibition of cancer cell proliferation [185]. Notably, direct IL-6 administration could not mimic exercise-related anticancer effects, implying that the role of IL-6 required a prior exercise-induced activation and recruitment of immune cells.

### Activity–rest cycles regulate tumor microenvironment

Vascular remodeling attributed to abundant pro-angiogenic cytokines, such as VEGF (vascular endothelial growth factor) in the tumor microenvironment, is possibly the most well-known benefit of exercise on oncology indicated from pre-clinical investigations [227–230]. Numerous studies have revealed that vessel density and perfusion in tumors are involved in exercise-mediated VEGF production [231–233]. Furthermore, during exercise, endothelial cell recruitment is mediated by platelet derived growth factor receptor-beta (PDGFRβ) derived from platelets for tumor angiogenesis, dramatically reducing tumor hypoxia by upregulation of micro-vessel density and perfusion [232, 234, 235]. Hypoxia usually stimulates several stress response pathways to induce changes in the tumor microenvironment, especially immune microenvironment remodeling, therefore promoting tumor development [236–238]. For example, cytokines IL-4 and IL-10 triggered by hypoxia can induce differentiation of tumor-associated macrophages (TAMs) into an immunosuppressive M2 phenotype [239, 240]. In addition, the hypoxic environment stimulates dendritic cells to induce the expression of indoleamine 2,3-dioxygenase (IDO), which performs an immunomodulatory role in T cell suppression [241–243]. A previous study also found reduction of CD8^+^ tumor-infiltrating lymphocytes (TILs) presenting in tumor hypoxic regions [244, 245]. A hypoxic microenvironment has also been indicated to contribute to both dendritic cells-mediated inactivation of TILs and abnormal expression of PD-L1, resulting in immunotherapy resistance [246–249]. Interestingly, hyperoxia (60% oxygen) produced more than three-fold tumor infiltration CD8^+^ TILs compared to the control mice [244]. This finding provides the potential clinical implications of oxygen content regulation as every 10% increase in CD8 + TILs results in a 19% decrease in patient mortality [250]. However, sequestering chronic tumor patients in a 60% oxygen environment is not practical. Exercise, representing an available alternative, can reduce hypoxia in tumors, thus sensitizing cancer cells to immunotherapy by further increasing immune cell infiltration and reducing IDO-mediated immunosuppression.

Hippo signaling is one of the most important pathways for tumor initiation and development [251, 252]. This pathway involves the growth and differentiation of tumor cells, and regulation of several signaling pathways related to the formation of the tumor microenvironment (including extracellular matrix remodeling) [253, 254]. Recently, a study has reported that exercise was involved in the regulation of the Hippo signaling pathway [255]. Mechanically, exercise-driven epinephrine markedly inhibited tumor formation by inducing Yap and Taz phosphorylation and subsequent degradation [255]. In line with this, serum samples from breast cancer patients who were scheduled to participate in exercise training were able to show a 50% decrease in breast cancer metastasis in an experimental tumor model, attributed to exercise-induced catecholamine release [255].

Additional mechanisms are currently being investigated in exercise-mediated reduction of cancer progression. Secretion of several systemic factors (such as catecholamines, myokines), upregulation of blood flow-related shear stress, sympathetic activation, as well as increased body temperature, display immediate stress on tumor metabolism and homeostasis during exercise training [256]. If people are undertaking long-term training, the effects mentioned above will result in steady intratumor changes, such as strong immunogenicity, metabolism adaptation, and improved blood perfusion, which should help mitigate tumor development [257, 258].

## Potential application of clock-associated therapy in cancer management

**Fig. 5:**
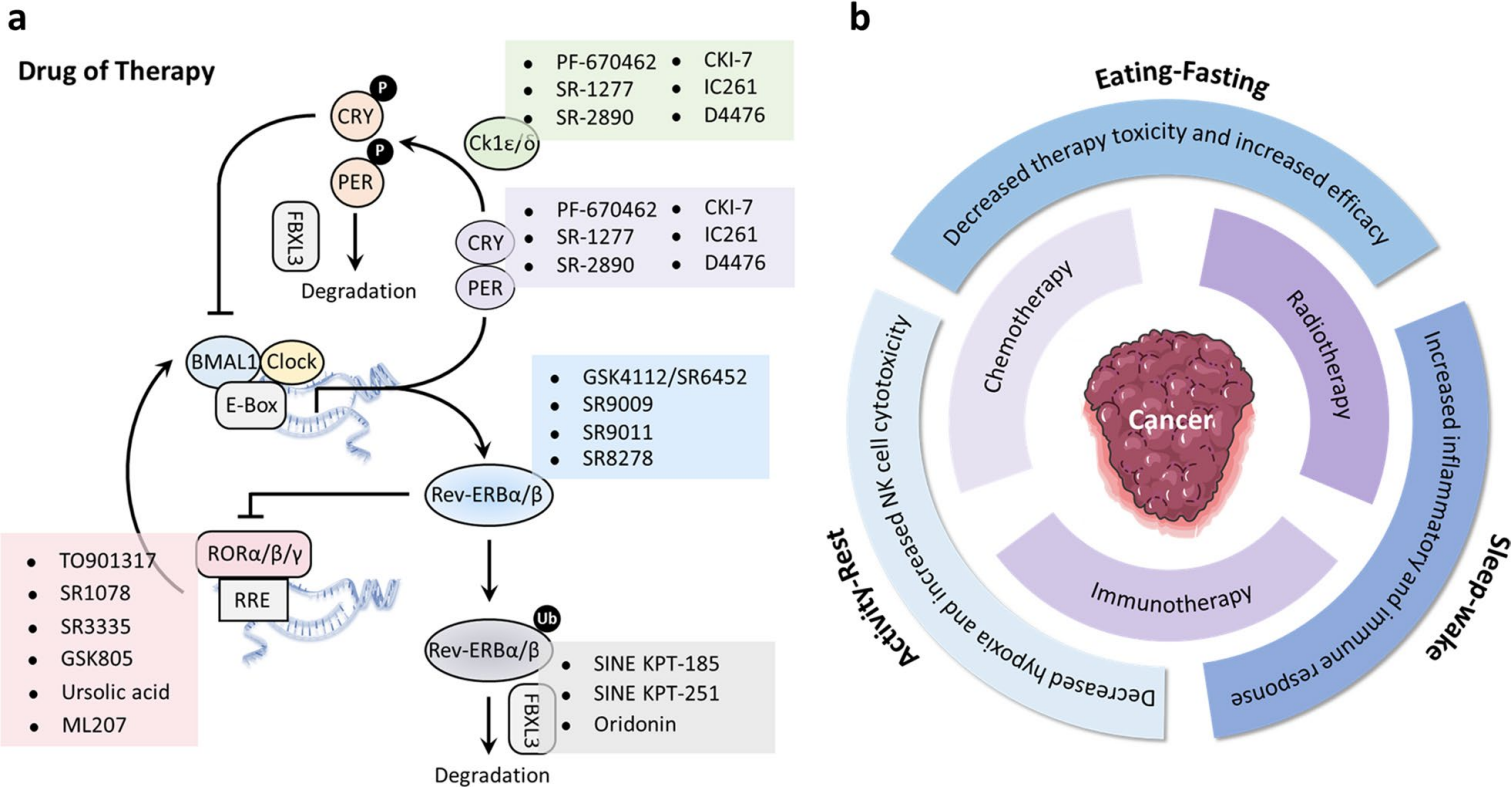
Clock-based therapy in cancer management. **a** directly pharmacological targeting circadian clock for cancer therapy. Targeting the components of circadian clock has attracted much attention as a therapeutic approach to treat cancer. There are several pharmacologic agents targeting the components of circadian clock, including REV-ERBα/β, RORα/β/γ, CRY1/2, Casein Kinase family, and FBXL3. **b**, the effect of modulating circadian rhythms on conventional cancer therapy. Modulating the sleep–wake, eating–fasting, and activity–rest cycles can benefit the effect of chemotherapy, radiotherapy, and immunotherapy

The molecular understanding of circadian rhythms has raised new therapeutic frontiers for cancer which could put the circadian clock in an indispensable treatment role. Therefore, pharmacological modulation of the circadian clock and/or treating cancer in the clock may hold potential as new therapeutic options for better cancer management (Fig. 5).

### Pharmacological targeting core components of the circadian clock for cancer therapy

Targeting the components of circadian clock has attracted much attention as a novel therapeutic approach to treat chronic diseases, such as chronic inflammatory diseases, metabolic syndrome, and cancer [259, 260]. Theoretically, there exist two drug approaches for targeting the circadian clock: either by directly modulating the core circadian genes, or targeting their regulators. However, as BMAL1 and CLOCK are transcriptional factors, it is notoriously challenging to directly target these circadian core genes [261, 262]. Hence, pharmacologic agents targeting proteins responsible for phosphorylation or degradation of clock components which negatively regulate BMAL1 and CLOCK have been developed as agonists or antagonists to disrupt the circadian network.

#### REV-ERBs

REV-ERBα/β are nuclear hormone receptors that can directly bind to the promoter of BMAL1 and CLOCK and thus negatively regulate their transcription [263–265]. The aberrant expression of REV-ERBs has been found in many cancer types in which they mainly regulate plasma glucose level, lipid, and energetic metabolism [266]. GSK4112, a small molecule molecular probe also known as SR6452, was the first synthetic agonist of REV-ERB obtained from a fluorescence resonance energy transfer biochemical screen, with an EC50 of 2.3 μM for inhibiting the transcriptional activity of *Bmal1* [267, 268]. However, the unsatisfactory pharmacokinetic profile and specificity of GSK4112 limited its use as a chemical tool in vivo, which drove the development of pyrrole derivatives SR9009 and SR9011 [269, 270]. These two different agonists of REV-ERBs have been reported to display anticancer activity against different tumor types, including leukemia, brain, colon, breast, and melanoma, while exhibiting no obvious side effect on normal cells or tissues [271–274]. Further studies found that autophagy and de novo lipogenesis were identified as the key events in evoking the apoptotic response in malignant cells treated with SR9009 and SR9011 [274]. In addition, SR9011 and SR9009 also reduced glioblastoma stem cell (GSC) proliferation and were lethal to chemoresistant small-cell lung cancer (SCLC) cells by repressing the expression of the tricarboxylic acid (TCA) cycle enzymes [275] and suppression of autophagy [273], respectively. Other chemical agonists for REV-ERBα, including GSK2945, GSK0999, GSK5072, and GSK2667 have also been developed but their effects on cancer need further investigation [276].

#### RORs

Unlike REV-ERBs, RORs (including RORα, RORβ, and RORγ) can constitutively induce the transcription of *Bmal1* through the ligand-independent recruitment of transcriptional co-activators [266, 277]. There is evidence showing that RORγ was upregulated in metastatic castrate-resistant prostate cancer and promoted the expression of the androgen receptor, thus raising the possibility of targeting RORγ for cancer treatment [278–282]. Notably, the ROR-γ-selective antagonists XY018 and SR2211 block the growth of prostate cancer cells with androgen receptor expression and restore sensitivity to enzalutamide treatment (a commonly used androgen inhibitor for prostate cancer), in which induction of apoptosis and decreased expression of key proliferation and survival proteins were identified as key events [278]. Moreover, RORγ was found to show important regulatory functions in pancreatic cancer stem cells, and pharmacological targeting of RORγ by SR2211 inhibits the tumor growth and prolongs survival in several in vivo pancreatic cancer models [283, 284]. In contrast, RORγ agonists can modulate multiple signaling pathways to enhance the antitumor immunity against leukemia, colon and breast cancer [285–288]. For example, LYC-54143 and LYC-53772 are two potent RORγ agonists which display cytotoxic activity by promoting cytokines/chemokines (i.e., IL-17A and GM-CSF) production and increasing co-stimulatory receptor expression (like CD226 and CD137) [288]. Based on this finding, another RORγ agonist, LYC-55716 (cintirorgon), is currently under Phase I clinical trial for treating patients with metastatic cancer alone [289] or in combination with pembrolizumab (clinical trial NCT03396497). Unlike RORγ, RORα was found to be downregulated in several cancer types, including breast, ovarian, and prostate cancer [290–293]. Treatment of RORα agonist SR1078 results in p53 stabilization and triggers apoptosis in human HepG2 cancer cells [294, 295]. Another study found that pevonedistat (MLN4924), a small molecule AMP mimetic, could stabilize RORα by inhibiting its ubiquitination and consequent degradation, which in turn attenuated cell proliferation of osteosarcoma, chondrosarcoma, and leukemia by inducing cell cycle arrest and apoptosis [296–299]. MLN4924 has been evaluated in 40 clinical trials related to cancer [300].

#### CRY1/2

Distinct from REV-ERBs, CRY1 and CRY2 are important for inhibiting clock-controlled gene transcription by directly interacting with BMAL1/CLOCK complexes [301–304]. KL001 was the first compound found to directly bind to and stabilize CRY by preventing FBXL3-mediated ubiquitin-dependent degradation [305]. A recent study has demonstrated that KL001 treatment decreased *OLIG2* and *SOX2* expression and inhibited GSC proliferation with reduced toxicity in normal brain cells [275, 306]. Interestingly, in vitro observations demonstrate that pharmacological targeting CRY by KS15 reduces proliferation of human breast cancer MCF-7 cells and increases the sensitivity of MCF-7 cells to treatment with doxorubicin and tamoxifen, but has no obvious cytotoxic effects on normal breast epithelial cells (MCF-10A) [307]. These contradictory findings indicate that the function of CRY in cancer treatment is context-dependent and that further research is needed to investigate its precise role.

#### Casein kinase (CK) family

The casein kinase 1 enzymes (CK1) are a family of serine/threonine kinases in which CK1δ and CK1ε phosphorylate PERs and CRYs and prime them for FBXL21 and β-TRCP mediated degradation [308–311]. The upregulation of CK1δ and CK1ε was observed in several cancer types, including melanoma, leukemia, pancreas, breast, and ovarian cancer, which highlights their potential as drug targets for anticancer therapy [312–315]. Indeed, breast cancer cells overexpressing CK1δ are sensitive to CK1δ/CK1ε inhibition caused by SR-3029 both in vitro and in vivo, while normal breast epithelial cells (MCF-10A) with low amounts of CK1δ are less sensitive to SR-3029 treatment [314, 316]. Another inhibitor, BTX-A51, was found to induce apoptosis in AML progenitor cells in vitro and suppress growth in vivo in both genetical-engineered AML mouse models and in patient-derived xenograft mouse models [317]. BTX-A51 is currently in Phase I clinical trials for the treatment of patients with relapsed or refractory AML (clinical trial NCT04243785). Similarly, CK2 (casein kinase II) has also been found to regulate cell growth and proliferation of several cancers [318]. For example, CK2α was upregulated in CRC cells and conferred resistance to 5-FU treatment by inhibiting ER stress-induced apoptosis, and treatment with a CK2α inhibitor may exert a synergistic effect with 5-FU against drug-resistant cancer cells [319]. Targeting CK2 with a pan-CK2 inhibitor, BMS-699, resulted in disruption of myeloid cell differentiation and increased efficacy of immunotherapy in mice with lung, colon and breast carcinoma and lymphoma [320]. Moreover, some potent CK2 inhibitors, such as CX-4945 (silmitasertib) and GO289, have been developed which inhibit the proliferation of several human tumors [321–323]. CX-4945 was the first CK2 inhibitor that entered into clinical trials for the treatment of human tumors [324, 325].

To sum up, several agonists or antagonists targeting the core components of circadian rhythm have been developed and show promising anticancer effect in various cancer types. However, few compounds are entering clinic trials to enable the evaluation of the true efficacy in patients, and the dependence of these anticancer effects on clock modulation still needs further investigation (Table 1).

**Table 1:** Summary of cancer types and their associations with disrupted rhythms

Cancer types	Disrupted rhythms	Promotion/inhibition	References
Breast cancer/prostate cancer	Shiftwork-induced disruption of sleep–wake cycles	Promotion	[49–52]
	Excessive calories-induced disruption of eating–fasting cycles	Promotion	[53, 54]
	Fasting-induced disruption of eating–fasting cycles	Inhibition	[140] [151]
	Exercise-induced disruption of activity–rest cycles	Inhibition	[183, 207–210]
Colorectal cancer	Short-term fasting-induced disruption of eating–fasting cycles	Inhibition	[149–151]
	Maintenance of activity–rest cycles	Inhibition	[55, 56]
	*Per2* mutation-induced disruption of circadian clock	Promotion	[57]
Lung cancer	*Per2* mutation-induced disruption of circadian clock	Promotion	[23]
	Fasting-induced disruption of eating–fasting cycles	Inhibition	[383]
	Exercise-induced disruption of activity–rest cycles	Inhibition	[412]
Liver cancer	*Per1/2* mutation or *Bmal1* deletion-induced disruption of circadian clock	Promotion	[60]
Acute lymphoblastic leukemia	Fasting-induced disruption of eating–fasting cycles	Inhibition	[167]
T cell lymphomas	*Cry1/2* or *Rorc* deletion-induced disruption of circadian clock	Promotion	[60, 61]
Glioma	Fasting-induced disruption of eating–fasting cycles	Inhibition	[141]
Pancreatic cancer	Fasting-induced disruption of eating–fasting cycles	Inhibition	[166]
	Exercise-induced disruption of activity–rest cycles	Inhibition	[426]

### Administrating drugs in clock: chronotherapy in cancer treatment

Chronotherapy has been defined as a strategy that utilizes the natural rhythms and cycles of physiological and biochemical processes to treat a disorder [326–328]. This type of therapy for cancer patients, aiming to reduce side effects and improve efficacy during cancer treatment, has been applied in the clinic even before more detailed mechanisms of the core circadian clock were known [329–332]. Subsequently, once the biological functions of circadian rhythms were revealed showing that drug PK, PD, and safety are highly depend on 24-h rhythm, the rationale for chronotherapy was supported [333–335]. Indeed, a suitable dosing time during the cycle can not only contribute to beneficial effects but also avoid adverse ones, especially for anticancer agents whose use may be limited due to their side effects on healthy host tissues [336–338]. For example, in cancer patients treated with constant-rate i.v. infusion of 5-fluorouracil (5-FU) for 5 days, the maximum plasma concentration (Cmax), and the best-tolerated time were found to be at 4:00 a.m [339, 340]. Recent reports have indicated circadian characteristics of inhibitors targeting the estrogen receptor and tyrosine kinases in both treated mice and patients, as evidenced by observed daily pharmacokinetic variations [341–344]. In addition, several Phase I to Phase III clinical trials have confirmed the efficacy of chrono-modulated treatment in various tumors [345, 346].

Importantly, chronotherapy potentially improves the survival rate and life quality of cancer patients by minimizing anticancer agents-driven cytotoxicity [347–349]. Mechanically, numerous anticancer drugs have consistently been demonstrated to display increased cytotoxicity to cells at specific phases of cell division, suggesting that optimization of dosing time for treatment by predicting circadian rhythms-related medicinal properties may translate into desired clinical outcomes [333, 338]. In a randomized trial, cancer patients with a sinusoidal chronotherapy schedule showed better tolerability and efficacy to drugs than with a constant-rate infusion [350]. Another study on 186 metastatic colorectal cancer patients in a randomized multicenter phase III trial also reported that oxaliplatin, 5-FU, and chronoFLO delivered by chrono-modulated infusion reduced ~ fivefold the rate of severe mucosal toxicity and ~ 50% functional impairment from peripheral sensitive neuropathy compared with constant drug delivery (14% vs. 76% and 16% vs. 31%, respectively) [329]. Furthermore, timed administration of irinotecan was performed in 31 cancer patients and demonstrated that chrono-mediated infusion of irinotecan from 2:00 a.m. to 8:00 a.m. induced less severe diarrhea and interpatient variability compared with the conventional 30-min infusion in the morning [351]. A very recent clinical study evaluated the effect of immunotherapy time-of-day infusion on the overall survival of patients with advanced melanoma. These results suggested that adaptive immune responses are less robust when infused in the evening than in the daytime [352, 353].

The application of a chronotherapeutic strategy for cancer treatment has stimulated developments in bioengineering, such as non-implantable multichannel time-programmable pumps for chrono-modulated drug delivery [354–356]. For example, in North America and the European Union, the IntelliJect™ device with four 30-mL reservoirs has been approved for treating cancer patients resulting in increased safety and efficacy of anticancer agents, based on chrono-mediated combination drug delivery of 5-FU, oxaliplatin, and leucovorin [328, 336, 357, 358]. The liver is a well-known rhythmic organ. However, liver having tumor metastases shows no marked circadian regulation. When anticancer drugs were infused in a chrono-modulated way directly into the hepatic artery of patients with liver metastases beneficial effects were observed [359, 360]. Indeed, this strategy has been confirmed as a safe and effective therapy by an international trial [361]. This study demonstrated that an automatic multichannel programmable pump, Mélodie®, could be used for treating colorectal cancer liver metastases by directly delivering anticancer agents (5-FU, oxaliplatin, irinotecan, and systemic cetuximab) into the hepatic artery, contributing to improvements in both toxic tolerability and drug effects [359, 361].

The emerging field of nanocarrier-mediated drug delivery on chronotherapy is now attracting increasing attention as it has been shown to increase cancer curability without added side effects and risks for the patients, as well as having potential cost savings [362–364]. An increasing body of data is showing that chronotherapy combined with nanotechnology offers important advantages for effective drug delivery, contributing to more efficient and safer cancer therapy [356, 365–367]. Recently, the synergistic anticancer effects of chrono-modulated delivery of paclitaxel (PTX)-loaded polymeric nanoparticles (PTX-NPs) were investigated on a human lung cancer-derived mouse xenograft model to identify the best time for drug delivery [368–370]. PTX in nanocarriers was initially rapidly released but subsequently turned into sustained release, showing a better anticancer effect than PTX single treatment [368]. Furthermore, the efficacy of PTX-NPs displayed a time-dependent effect and peaked at 15 h after light onset. Mechanically, PTX-NPs combined chronotherapy was verified to be associated with decreased Ki-67-mediated proliferation and CD31-mediated micro-vessel density, thereby resulting in inhibition of lung cancer by inducing cell apoptosis [368]. In addition to the above findings, a growing number of novel chrono-pharmaceutical delivery technologies are underway development and study. These strategies, by utilizing bedside or ambulatory pumps, facilitate more proficient cancer treatment by precisely delivering antitumor drugs in a circadian time-dependent manner.

In summary, chronotherapy is showing benefits not only for improving therapeutic efficiency but also for minimalizing the side effects of cancer treatment. However, the real application of chronotherapy in cancer treatment is still in its infancy, and more clinical trails are required to expand the clinical applications of chronotherapy in cancer treatment.

### Fasting in cancer prevention and treatment

The fasting-feeding pattern is an important time cue that can also determine the robustness of daily circadian rhythms [371–374]. Although maintaining a robust fasting-feeding pattern is correlated with prevention of tumorigenesis, disruption of this circadian rhythm, either by time/calorie-restricted feeding or intermittent/periodic fasting, may benefit the outcome of cancer treatment [375, 376]. For example, chronic calorie restriction (CR) and periodic fasting is attracting increasing attention for potential application to clinical cancer prevention and therapy based on long-lasting epidemiological studies and preclinical reports [159, 377–381]. A diet that mimics the effects of fasting (FMD) has been developed and used in a pilot clinical study, aiming to maintain the cancer-preventive effects from CR or fasting while reducing adverse effects [162, 382].

To date, four feasibility studies regarding cancer patients undergoing chemotherapy treated with fasting have been published [383–386]. In one of these studies, 10 patients with various cancers, such as lung, breast, esophageal, prostate, uterus, and ovarian cancer, volunteered to fast for 140 h before chemotherapy or for 56 h following chemotherapy. The results indicated that fasting induced no obvious adverse effects except for hunger and lightheadedness. Notably, 6 fasting patients showed a marked decrease in gastrointestinal, fatigue, and weakness events following chemotherapy. Furthermore, fasting did not compromise the anticancer effects from chemotherapy in those patients, as evidenced by no distinct difference of tumor volume or malignant markers compared to patients without fasting [383]. In line with this, 13 HER2-negative breast cancer patients were scheduled for treatment with fasting (only water supply) for 24 h or with standard nutrition regimens before and after the treatment with neo-adjuvant taxotere, adriamycin, and cyclophosphamide chemotherapy. Good tolerance was displayed to the short-term fasting, while a significant reduction of the erythrocyte and thrombocyte counts was shown at 7 days after chemotherapy [384]. Interestingly, non-fasted patients showed the upregulated expression of γ-H2AX (which indicates the levels of DNA damage) in leukocytes in 30 min after chemotherapy rather than the fasted patients [384]. Another interesting study evaluated the influence of fasting time with cancer patients. In this study, 20 patients primarily diagnosed with breast or ovarian cancer were randomized to treatment with 24- or 48-h fasting before platinum-derived chemotherapy or another 24-h fasting after platinum-derived chemotherapy (total 72-h fasting) [385]. During the fasting, three or more out of six patients in each cohort needed to consume less than 200 kcal per day. Fasting-related toxicities were only relatively minor, including dizziness, fatigue, and headache with some evidence of neutropenia in the 48- and 72-h cohorts [385]. Recently, a randomized clinical trial on 34 patients with ovarian or breast cancer was conducted to assess the effects of an FMD on life quality and side effects of chemotherapy [386]. In this study, a 36 ~ 48-h limited intake of 400 kcal per day was required before the treatment of chemotherapy. Subsequently, the same calorie limitation was performed for a further 24 h following chemotherapy [386]. From these data we can conclude that FMD does not lead to serious side effects per se, while improving chemotherapy-induced adverse reaction, like fatigue.

Therefore, fasting, either periodic or short-term, is attracting increasing attention for clinical cancer prevention and therapy and indeed displays visible effect on cancer treatment in some clinic trails, suggesting a potential adjuvant therapy for cancer in the future.

### Exercise benefits cancer therapy

Timing exercise has long been utilized as a Zeitgeber for regulating circadian clock of some major body systems, such as skeletal muscle and the blood vessels [387–391]. Maintaining this clock might be an efficacious strategy for preventing and combating metabolic disease, including obesity, diabetes, cardiovascular disease, and cancer [392–394].

Accumulating literature has shown that exercise training can not only relieve treatment side effects but may also hold the potential to increase the potency and efficacy of cancer therapies [256, 395–397]. Emerging evidence from pre-clinical studies has indicated that exercise regulates intratumoral vascular perfusion, systemic inflammation, sex hormone levels, and immune cell function during cancer treatment, implying that exercise may be not only healthy but also therapeutic [398–400].

The first line of treatment for most solid tumors is surgery, and radical tumor resection is usually considered as the most efficient therapeutic strategy, especially if the tumor is detected before it has metastasized [401]. Exercise training has been proved to be beneficial for the management of operable tumors at several distinct time points along the surgical treatment period, including the preoperative period, short-term immediately after surgery, and longer-term follow up [402–405] and is becoming increasingly popular. For example, in patients with early-stage lung cancer, preoperative exercise was found to improve walking endurance, peak exercise capacity, dyspnea and postoperative pulmonary complications, which are associated with the time of intensive care admissions, hospital stays, and hospital readmissions, as well as mortality in these patients [406]. Indeed, data from systematic review and meta-analysis have demonstrated that lung cancer patients undergoing an exercise regime had a lower risk of postoperative pulmonary complication, fewer days of intercostal catheter use, and a shorter length of hospitalization when compared with non-exercise patients [407–409]. In addition, exercise training immediately after cancer surgery is also beneficial to the outcome of cancer patients. Studies have been conducted to evaluate the effectiveness of an early onset exercise program on shoulder mobility, physical performance, and postoperative complications of breast cancer patients and found that shoulder-arm mobility of surgical breast cancer patients demonstrated significant improvement compared with the control group [410, 411]. Moreover, longer-term exercise following surgery has been established as a feasible, safe, and effective method of decreasing postoperative side effects and improving life quality in lung cancer patients [412]. Nevertheless, the mechanisms behind the effects of exercise on surgical outcomes are still poorly understood, and further studies are needed to clarify the potential of exercise training for better cancer management in the perioperative period.

Radiotherapy is a cornerstone for cancer management, the effectiveness of which is dependent on sufficient delivery of oxygen to the tumor sites to generate enough ROS for eliminating cancer cells [413, 414]. Exercise training is a promising adjunct therapy for cancer patients receiving radiotherapy, mainly by affecting blood circulation and oxygen delivery to the tumor location [415]. Several randomized controlled trials have reported that exercise training could mitigate fatigue in cancer patients receiving radiotherapy and generate longer-term improvements and additional benefits for quality of life [416, 417], as well as reducing the severity of rectal toxicity during radiotherapy of prostate cancer [418]. Moreover, evidence from a preclinical mouse model of prostate cancer also demonstrated that exercise can improve radiotherapy efficiency by increasing natural killer cell infiltration and activity, resulting in increased tumor cell apoptosis [419]. However, very few clinical studies have been conducted to investigate the effect of either acute or prolonged exercise training on radiotherapy treatment response, thus delaying the application of exercise training in clinical cancer treatment.

Likewise, the efficacies of chemotherapy and immunotherapy are partially dependent on adequate intratumoral blood perfusion which can be enhanced by appropriate exercise training to assist in delivery of drugs or immune cells to the tumor sites [420]. Several preclinical studies using mouse models have found that soft aerobic exercise promotes tumor vascular function to enhance drug delivery to breast, prostate, and pancreatic tumors, thus increasing chemotherapy efficacy [421–425]. Moreover, exercise has been demonstrated to remodel the tumor vasculature in a PDX mouse model, which improves tumor regression and inhibits the recurrence of pancreatic ductal adenocarcinoma (PDAC) under gemcitabine treatment [426]. A previous study found that NK cell infiltration was significantly increased in tumors in wheel-running treated mice. Mechanistic analyses demonstrated that the growth suppression of tumors was attributed to an epinephrine-IL6-dependent mobilization and activation of NK cells [185]. Another recent study found that the combination of anti-PD-1 and exercise increased the percentage of CD8^+^ T cells and enhanced antitumor immune responses in mice with EO771 breast tumors [249]. However, much remains to be understood about the efficiency of exercise on immunotherapy in clinical cancer management.

Overall, the above findings indicate the potential therapeutic role of exercise training in cancer treatment. Nevertheless, current insights into the mechanistic effects of exercise on cancer therapy mainly stem from pre-clinical studies. Additional studies, especially a further understanding of the mechanisms involved and how they are controlled together with targeted clinical trials, are essential for a deeper understanding of the synergistic effects of exercise and conventional anticancer therapies.

## Conclusions and perspectives

As discussed above, circadian control of biological processes is involved in almost all aspects of daily life, and tightly regulates multiple human behaviors, including sleep–wake, eating–fasting and activity–rest cycles. Chronic disruption of these biorhythms induced by external environmental or physiological changes may contribute to a wide range of human diseases, including sleep disorders, psychiatric and neurodegenerative diseases, cardiovascular disease, and especially cancers [11, 326, 427–430]. Indeed, several epidemiologic and animal studies have demonstrated the important role of the circadian clock in tumorigenesis and progression [427]. Although gene expression, cell division, and DNA repair are modulated by the clock, the concept that clock genes are general tumor suppressors remains unproven [326]. Moreover, tumor development will clearly cause clock disruptions, such as sleep deprivation and insufficient food intake, affecting bodily functions. This Janus effect is therefore attracting considerable interest worldwide for developing novel therapeutic strategies by modulating circadian rhythms. Table 2 summarizes the clinical studies related to clock-associated cancer therapy.

**Table 2:** Summary of clinical studies in relation to circadian-modulated cancer therapy

Treatments/drugs (targets)	Study design	Cancer type	Main findings	Ref
LYC-55716 (RORγ agonist)	Phase 1 clinical trials treating patients with LYC-55716 alone or in combination with pembrolizumab	Relapsed metastatic cancer (NSCLC, gastroesophageal, renal cell, urothelial, and ovarian)	Confirmed its safety and tolerability and choice of 450 mg BID dose for a phase 2a study	[289]
MLN4924 (RORα stabilization)	A phase 1b study of MLN4924 plus docetaxel, gemcitabine, or combination of carboplatin and paclitaxel	Solid tumors	MLN4924 with docetaxel or with carboplatin plus paclitaxel was tolerable without cumulative toxicity	[435]
	Phase 1, dose escalation study of MLN4924 in adult patients	Lymphoma and multiple myeloma	The population pharmacokinetic of MLN4924 was profiled	[436]
BTX-A51 (CKIα inhibitor)	Phase 1a study to evaluate the safety, toxicity, pharmacokinetics and preliminary efficacy	Relapsed or refractory acute myeloid leukemia	Ongoing	/
Chrono-modulated capecitabine	Phase 2 study to evaluate the feasibility and tolerability of capecitabine according to a specific time schedule	Locally advanced primary rectal cancer	Chrono-modulated capecitabine combined with adjuvant radiation therapy is tolerated and feasible	[345]
Chronotherapy with oxaliplatin, fluorouracil, and folinic acid	Phase 3 study to test chrono-modulated infusion of oxaliplatin, fluorouracil, and folinic acid compared with a constant-rate infusion method	Metastatic colorectal cancer	Chronotherapy was significantly less toxic and more effective than constant-rate infusion	[329]
Chrono-modulated infusion of 5-fluorouracil and 5-fluorodeoxyuridine	Randomized phase II trial comparing flat versus chrono-modulated infusion	Hepatic metastases from colorectal cancer	Supporting the use of chronotherapy in treating colorectal cancer liver metastases with combined arterial and venous fluoropyrimidine chemotherapy	[437]
Chrono-modulated infusion of 5-fluorouracil and mitomycin-C	Phase 3 study of mitomycin-C with protracted venous infusion or circadian-timed infusion of 5-fluorouracil	Advanced colorectal carcinoma	Dose intensification of 5-FU using a circadian-timed infusion did not lead to improved response or survival	[438]
Vinorelbine combined with chrono-modulated 5-fluorouracil	Phase 1 trial to study the optimal circadian time of vinorelbine combined with 5- fluorouracil	Metastatic breast cancer	Future optimal time-finding trials should have tolerability and/or activity as the primary endpoint in place of a particular toxicity	[439]
4'-O-tetrahydropyranyl doxorubicin and cisplatin	Randomized phase 2 trial to evaluate the circadian timing and dose-intensity	Advanced ovarian cancer	Dosing doxorubicin in the early morning and cisplatin in the late afternoon could decrease toxicities	[434]
Sunitinib	Phase 2 study of sunitinib administered in a continuous once-daily dosing regimen	Metastatic renal cell carcinoma	Administration of 37.5 mg sunitinib on a continuous once-daily dosing regimen has a manageable safety profile	[440]
Chrono-modulated infusion of oxaliplatin in combination with capecitabine	A randomized phase 2/3 trial to compare short-time and chrono-modulated infusion of oxaliplatin in combination with capecitabine	Advanced colorectal cancer	Infusion of 30-min oxaliplatin is safe and does not increase the severity of chronic neuropathy	[441]
Cisplatin-based chronotherapy	A randomized controlled study to evaluate the superiority of cisplatin-based chronotherapy and pharmacokinetics for cisplatin	Advanced non-small cell lung cancer	Cisplatin-based chronotherapy has advantage in relieving side effects of chemotherapy	[442]
Chronotherapy of interleukin-2	A phase 1/2 study to evaluate the safety of IL-2 chronotherapy and determine the maximum tolerated dose	Metastatic renal cell carcinoma	IL-2 chronotherapy is safe, moderately toxic and active in metastatic RCC	[443]
Chrono-modulated capecitabine treatment	A phase 1 study to evaluate the pharmacology of continuous chrono-modulated capecitabine treatment	Advanced solid tumors	Chrono-modulated treatment with capecitabine can lead to improved tolerability and efficacy	[444]
Cetuximab and circadian chrono-modulated chemotherapy	A clinical trial to study safety, efficacy and improved secondary surgical resectability of combinational treatment	Metastatic colorectal cancer	Cetuximab combined with chronotherapy shows safe and effective therapeutic control of metastases	[445]
Chrono-modulated treatment of oxaliplatin, 5-fluorouracil and sodium folinate	A clinical trial to evaluate non-hematological toxicity and patient characteristics treated with chrono-modulated chemotherapy	Metastatic gastrointestinal cancer	The chrono-modulated regimen with oxaliplatin, 5-FU and sodium folinate displays a manageable toxicity which depends on the patient characteristics	[446]

There are, however, some concerns that need to be addressed before the successful application of combining conventional treatment with targeting of the circadian clock for clinical cancer management. Firstly, targeting one of the clock components may prove no more effective than combinational therapeutic strategies using two or more targeted drugs. Secondly, the circadian clock can coordinate disparate cellular pathways. This may result in unwanted side effects or even opposite outcomes during treatment. Therefore, determining the appropriate dosage to successfully target circadian rhythm to obtain transient repression rather than chronic treatment may be required to avoid detrimental effects. Most importantly, the preclinical efficacy of manipulating circadian rhythms needs to be extended to larger study cohorts [327, 431], as results from a previous study with a small sample size claiming a substantial effect of chronotherapy on ovarian cancer were not reproduced in subsequent larger studies [432–434].

In summary, circadian rhythms participate in diverse biological pathways. Their manipulation holds the promise for beneficial treatment of not only cancer, but also other serious diseases. Based on significant advances in this area, which was honored by the 2017 Nobel Prize for Physiology or Medicine awarded jointly to Jeffrey C. Hall, Michael Rosbash and Michael W. Young, we believe that it will have excellent clinical potential in the near future.

## Data Availability

Not applicable.

## Abbreviations

AML: Acute myeloid leukemia
BMAL1: Brain and muscle aryl hydrocarbon receptor nuclear translocator 1
CKIε: Casein kinase Iε
CLOCK: Circadian locomotor output cycles kaput
CR: Calorie restriction
CRC: Colorectal cancer
CRP: C-reactive protein
DEN: Diethylnitrosamine
DSR: Differential stress resistance
DSS: Differential stress sensitization
ENT1: Equilibrative nucleoside transporter 1
FMDs: Fasting-mimicking diets
GH: Growth hormone
GSC: Glioblastoma stem cell
HDACs: Histone deacetylases
IARC: International Agency for Research on Cancer
IDO: Indoleamine 2,3-dioxygenase
IFN: Interferon
LAK: Lymphokine-activated killer
LID: Limited ingredient diet
NK: Natural killer
NO: Nitric oxide
NREM: Non-rapid eye movement
OSM: Oncostatin M
PDAC: Pancreatic ductal adenocarcinoma
PDGFRβ: Platelet derived growth factor receptor-beta
PRDM1: Protein PR/SET domain 1
PRL: Prolactin
REM: Rapid eyes movement
RORs: Subfamilies of nuclear hormone receptors
ROS: Reactive oxygen species
SCLC: Small-cell lung cancer
SCN: Suprachiasmatic nucleus
sICAM: Soluble intercellular adhesions molecule
SPARC: Secreted protein acidic and rich in cysteine
STF: Short-term fasting
SWS: Slow-wave sleep
TAMs: Tumor-associated macrophages
TCA: Tricarboxylic acid
TILs: Tumor-infiltrating lymphocytes
TKIs: Tyrosine kinase inhibitors
TLR4: Toll-like receptor 4
VEGF: Vascular endothelial growth factor
WHO: World Health Organization
